# Case Report: Whole-body electrical muscle stimulation as an adjunctive tool in cardiac rehabilitation of a patient with heart failure and reduced ejection fraction

**DOI:** 10.3389/fcvm.2026.1863805

**Published:** 2026-07-08

**Authors:** Damian Sendrowski, Agata Polańska-Szczap, Beata Hus, Anastasiia Vlaieva, Szymon Markowski, Abraham Carlé-Calo, Dariusz Kozłowski

**Affiliations:** 1 Institute of Health Sciences, Pomeranian University in Słupsk, Słupsk, Poland; 2 Department of Cardiology, Janusz Korczak Voivodeship Regional Specialist Hospital in Słupsk Ltd., Słupsk, Poland; 3 Wiemspro Polska, Jaworze, Poland; 4 Department of Physiology, Faculty of Medicine, University of Granada, Granada, Spain; 5 Wiemspro EMS (Wiemspro S.L.), Málaga, Spain; 6 Department of Cardiology and Electrotherapy, Faculty of Medicine, Medical University of Gdańsk, Gdańsk, Poland

**Keywords:** cardiac rehabilitation, CARE guidelines, case report, electrical muscle stimulation, heart failure, physiotherapy, reduced ejection fraction, whole-body EMS

## Abstract

**Background:**

Whole-body electrical muscle stimulation (WB-EMS) is an emerging modality that simultaneously activates multiple large muscle groups via a wearable electrode suit. Although localized neuromuscular electrical stimulation (NMES) has demonstrated efficacy as an adjunctive strategy in cardiac rehabilitation (CR), WB-EMS has not previously been investigated in patients with heart failure and reduced ejection fraction (HFrEF) undergoing CR.

**Case description:**

A 73-year-old male with recurrent non-ST-elevation myocardial infarction (NSTEMI), chronic heart failure (LVEF 20%–25%), and New York Heart Association class III was admitted for a 24-day inpatient CR programme. Standard CR (cycle ergometry, treadmill, resistance exercises, respiratory physiotherapy, and education, five days/week) was augmented with WB-EMS (Wiems Revolution Pro; 85 Hz bipolar, 350 μs, 4 s on/4 s off, 20 min sessions) three times per week (ten sessions). Safety was monitored with serial creatine kinase (CK), high-sensitivity troponin I (hs-TnI), N-terminal pro-brain natriuretic peptide (NT-proBNP), C-reactive protein (CRP), and venous blood gas with lactate, measured before and 2–3 h after each session.

**Outcomes:**

A modest, age-appropriate improvement in functional capacity was accompanied by substantial reverse left-ventricular remodelling: LVEF improved from 27% to 54% (Simpson biplane); peak workload from 66 to 97 W (+47%); six-minute walk test distance from 390 to 590 m (+51%); peak oxygen uptake from 17.42 to 19.02 mL/min/kg (+9.2%); and EQ-5D-5L visual analogue scale from 60 to 90/100. Pre-exercise NT-proBNP fluctuated across the ten sessions (range 318.3–1,019.3 pg/mL); the final-day rise to 1,019.3 pg/mL likely reflected greater training effort on the last exercise day, supported by the concurrently highest post-session venous lactate of the programme (4.4 mmol/L; *Δ* + 2.9 mmol/L). One-month International Physical Activity Questionnaire data demonstrated sustained increases in daily physical activity. CK, hs-TnI, CRP, and venous lactate remained within acceptable safety limits, with no clinical adverse events attributable to WB-EMS.

**Discussion:**

To our knowledge, this is the first report of WB-EMS combined with standard CR in an HFrEF patient. Comprehensive serial biomarker monitoring confirmed a favorable safety profile. Improvements across functional, echocardiographic, and quality-of-life domains are promising but require confirmation in randomized trials, as multiple concurrent therapeutic interventions preclude causal attribution to WB-EMS alone.

## 1 Introduction

Heart failure with reduced ejection fraction (HFrEF) remains a leading cause of morbidity and mortality worldwide. Exercise-based cardiac rehabilitation (CR) is a Class I recommendation for patients with HF and significantly improves exercise capacity, quality of life, and the risk of hospitalization (1, 2). Despite robust evidence, uptake of CR remains low, particularly among frail elderly patients with advanced disease and severe deconditioning who cannot fully engage in conventional exercise programmes.

Neuromuscular electrical stimulation (NMES) has emerged as an adjunctive strategy for patients with limited exercise tolerance. A meta-analysis demonstrated that NMES significantly improves peak oxygen consumption, six-minute walk test (6MWT) distance, quality of life, and peripheral muscle strength in HF (3). More recent randomized controlled trials have confirmed these benefits in frail elderly populations, including a home-based NMES crossover trial in older adults with chronic HF (4) and the ACTIVE-EMS trial in acute HF (5). A companion systematic review by our group (6) confirmed that peripheral muscle electrostimulation is safe and feasible alongside conventional CR in frail elderly cardiac patients, improving peripheral muscle function without cardiovascular stress.

Whole-body electromyostimulation (WB-EMS) differs from localized NMES in that it simultaneously activates multiple large muscle groups — upper arms, chest, back, abdomen, gluteal region, and thighs — via a wearable electrode suit. WB-EMS has shown benefits in non-athletic adults across a range of health-related outcomes, including body composition, muscle strength, and cardiorespiratory fitness (7). However, to our knowledge, WB-EMS has not previously been investigated in patients with HFrEF undergoing CR, representing a significant gap in the evidence base.

This report presents the first application of WB-EMS as an adjunct to standard inpatient CR in an HFrEF patient, drawn from the active arm of an ongoing randomized controlled trial. The case is reported in accordance with the CARE guidelines (8). The primary aim is to describe the safety profile and clinical outcomes of this novel intervention in order to provide proof-of-concept data for the ongoing trial.

## 2 Case description

### 2.1 Patient presentation

A 73-year-old male (height 170 cm, weight 90 kg, body mass index 31.1 kg/m^2^) was admitted to the inpatient CR department on the day of CR admission, following revascularization for recurrent NSTEMI. His cardiac history included an initial NSTEMI approximately 17 years prior managed with percutaneous coronary intervention (PCI) of the right coronary artery (RCA) with a drug-eluting stent (DES). The index event was a recurrent NSTEMI approximately three weeks before CR admission, treated with PCI of the left anterior descending artery (LAD) with two DES, followed by staged PCI of the RCA with one DES approximately five days later (still during the index Cardiology admission). The hospital course was complicated by *Staphylococcus aureus* bacteraemia, treated with cloxacillin.

Comorbidities included chronic HF with severely reduced LVEF (20%–25%), NYHA class III (downgraded from IV during the acute episode), arterial hypertension, type 2 diabetes mellitus with vascular complications, and frequent premature ventricular complexes (PVCs) with non-sustained ventricular tachycardia (NSVT), prompting initiation of amiodarone during the index admission. Guideline-directed medical therapy (GDMT) optimization during the index hospitalization on the Cardiology Department followed the 2023 European Society of Cardiology (ESC) focused update on the diagnosis and treatment of HF (9): pre-admission beta-blocker (nebivolol 5 mg once daily) was switched to metoprolol succinate 47.5 mg twice daily (≈95 mg/day) on day 6, pre-admission spironolactone 25 mg once daily was switched to eplerenone 25 mg once daily on day 6, an angiotensin-converting enzyme inhibitor (perindopril) was switched to sacubitril/valsartan 97/103 mg twice daily on day 8, and empagliflozin 10 mg once daily was continued throughout. Loop diuresis was intensified with furosemide 40 mg once daily and torasemide 10 mg once daily; amlodipine 10 mg once daily was switched to lercanidipine on day 9 (peripheral oedema) and titrated to lercanidipine 5 mg once daily at discharge. Lipid-lowering therapy was intensified from rosuvastatin monotherapy to a fixed-dose combination of rosuvastatin 20 mg + ezetimibe 10 mg once daily. Antiarrhythmic therapy comprised amiodarone (intravenous 300 mg on day 9, oral loading 600 mg/day on days 7–15, then maintenance 200 mg/day from day 16). Antiplatelet therapy comprised acetylsalicylic acid 75 mg once daily continued throughout, with prasugrel 60 mg loading on day 1 then 10 mg once daily maintenance. Metformin 1,000 mg twice daily was initiated on day 21 (at admission to the Cardiac Rehabilitation Department) and pantoprazole 20 mg once daily provided gastroprotection during dual antiplatelet therapy. A detailed admission-to-discharge medication table is provided in the (Supplementary Table S1). A timeline of the key clinical events, from the initial NSTEMI to the one-month follow-up, is summarized in Table 1.

**Table 1:** Timeline of key clinical events.

Time-point	Event
∼17 years prior	Initial NSTEMI; PCI of RCA with DES
∼3 weeks before CR admission	Recurrent NSTEMI; PCI of LAD with 2× DES
Index Cardiology admission	Staged PCI of RCA with 1× DES
CR Day 1 (admission)	Admission to inpatient CR Department; baseline CPET
CR Day 2	Baseline echocardiography: LVEF 27%
CR Day 4 (Session 1)	Baseline laboratory assessments; first WB-EMS session
CR programme (Sessions 1–10)	Standard CR + WB-EMS programme (10 sessions)
CR Day 22	Discharge echocardiography: LVEF 54%
CR Day 24	Discharge CPET; 6MWT 590 m
CR Day 25 (discharge)	Discharge in good general condition; NYHA I
One month after discharge	One-month follow-up: substantially increased physical activity (IPAQ)

NSTEMI, non-ST-elevation myocardial infarction; PCI, percutaneous coronary intervention; RCA, right coronary artery; LAD, left anterior descending artery; DES, drug-eluting stent; CPET, cardiopulmonary exercise testing; CR, cardiac rehabilitation; WB-EMS, whole-body electrical muscle stimulation; LVEF, left ventricular ejection fraction; 6MWT, six-minute walk test; NYHA, New York Heart Association; IPAQ, International Physical Activity Questionnaire.

### 2.2 Baseline assessment

Baseline echocardiography (CR Day 2) revealed an enlarged left ventricle (end-diastolic diameter 67 mm, end-systolic diameter 55 mm) with severe global systolic dysfunction and dyssynchrony. LVEF was 27% by Simpson biplane (automated 23%). Global longitudinal strain was markedly impaired (two-chamber −10.9%; parasternal long-axis −5.5%). Mild mitral regurgitation was present. Right ventricular function was preserved [tricuspid annular plane systolic excursion (TAPSE) 23 mm, tricuspid regurgitation velocity 2.1 m/s, estimated right ventricular systolic pressure <36 mmHg].

Baseline cardiopulmonary exercise testing (CPET; the CR admission day) using a modified Bruce ramp protocol demonstrated peak workload 66 W (46% predicted), peak oxygen consumption (VO_2_max) 17.42 mL/min/kg, peak metabolic equivalents (METs) 5.0, and peak heart rate 132/min (90% predicted; respiratory exchange ratio 1.03). ECG monitoring documented frequent PVCs, bigeminy, couplets, and non-sustained ventricular tachycardia salvos, suppressed at peak exercise but increased during recovery. Pressor and chronotropic responses were excessive. Training parameters were set at heart rate 120–125/min and maximum workload 60–65 W. Baseline 6MWT distance was 390 m. The baseline EQ-5D-5L profile was 11,112 (slight anxiety), with EQ-VAS 60/100. Baseline International Physical Activity Questionnaire (IPAQ) responses indicated minimal physical activity.

Baseline laboratory values (CR Day 4, Session 1) were: NT-proBNP 544 pg/mL, hs-TnI 4.9 ng/L, CK 64 U/L, CRP 5.1 mg/L, creatinine 1.41 mg/dL (eGFR 49 mL/min/1.73 m^2^), fasting glucose 126 mg/dL, total protein 7.1 g/dL, albumin 4.0 g/dL, sodium 141 mmol/L, potassium 4.6 mmol/L.

### 2.3 Therapeutic intervention

Standard CR comprised supervised aerobic exercise (cycle ergometer and treadmill at training heart rate 120–125/min and maximum workload 60–65 W), resistance exercises, respiratory physiotherapy, and patient education, five days per week over 24 days (the 24-day CR admission).

**Figure 1:**
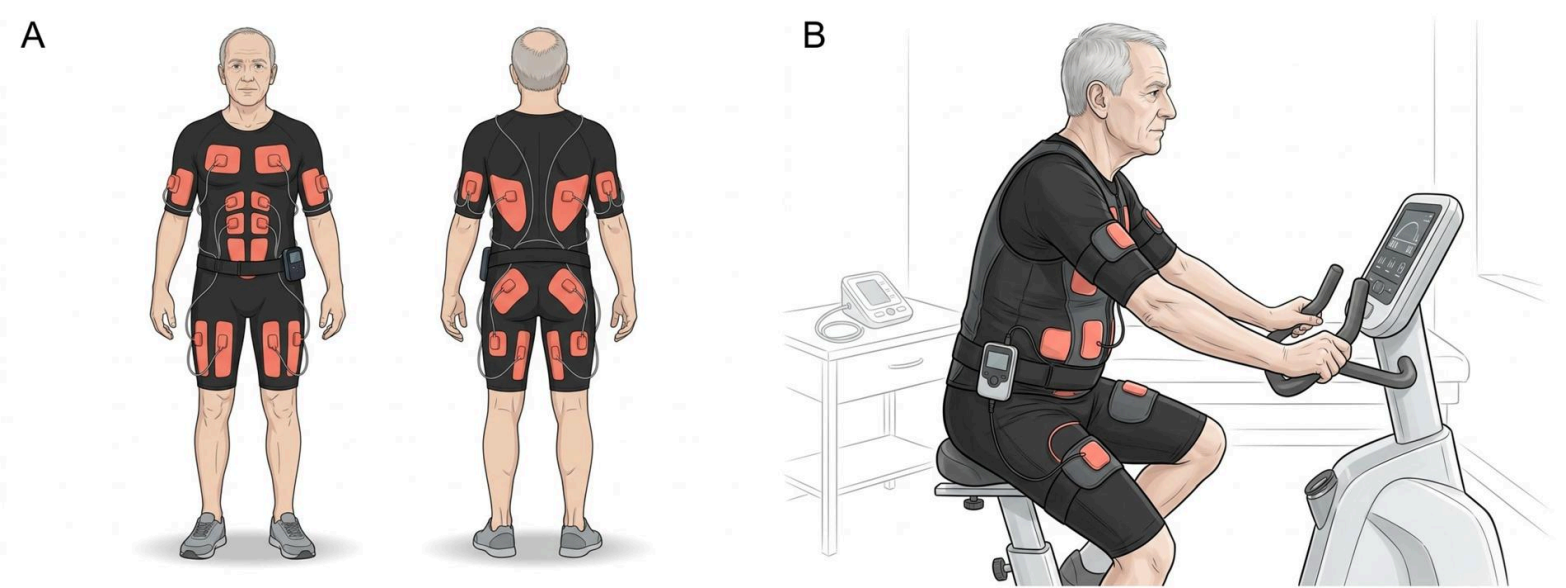
Equipment setup. **(A)** The Wiems Revolution Pro WB-EMS wearable electrode suit, showing positioning of bipolar electrodes over the major muscle groups (upper arms, chest, back, abdomen, gluteal region, and thighs) used for whole-body stimulation during cardiac rehabilitation sessions. **(B)** Representative cardiac rehabilitation session showing cycle ergometry performed while wearing the WB-EMS suit, as used during the 5 min warm-up that preceded each stimulation protocol. Illustrations created from clinical photographs to protect patient identity. Figures created by the authors; reproduced with written informed consent from the patient.

**Figure 2:**
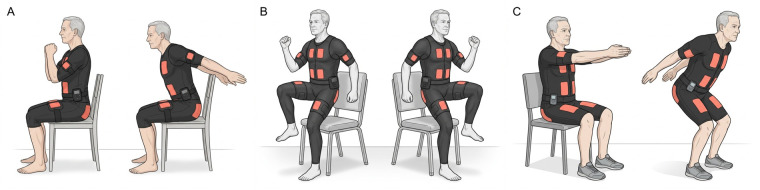
Seated exercises (Exercises 1–3 of the WB-EMS protocol). **(A)** Exercise 1 — Seated forward trunk inclination with upper-limb extension. The patient is seated upright; on the 4 s “on” phase he leans the trunk forward while simultaneously extending both arms forward, then returns to upright on the 4 s “off” phase. Targets paraspinal extensors, gluteal and core musculature with concurrent upper-limb activation. **(B)** Exercise 2 — Seated march. Performed in a seated position; alternating hip-flexion drives lift each knee in cadence with the duty cycle. Targets hip flexors, quadriceps, and core stabilizers with minimal weight-bearing demand. **(C)** Exercise 3 — Partial sit-to-stand. From the seated position the patient rises to a partial standing position then returns to seated, synchronized with the duty cycle. Targets the quadriceps, hamstrings, and gluteal muscles and serves as a transition to weight-bearing exercises.

**Figure 3:**
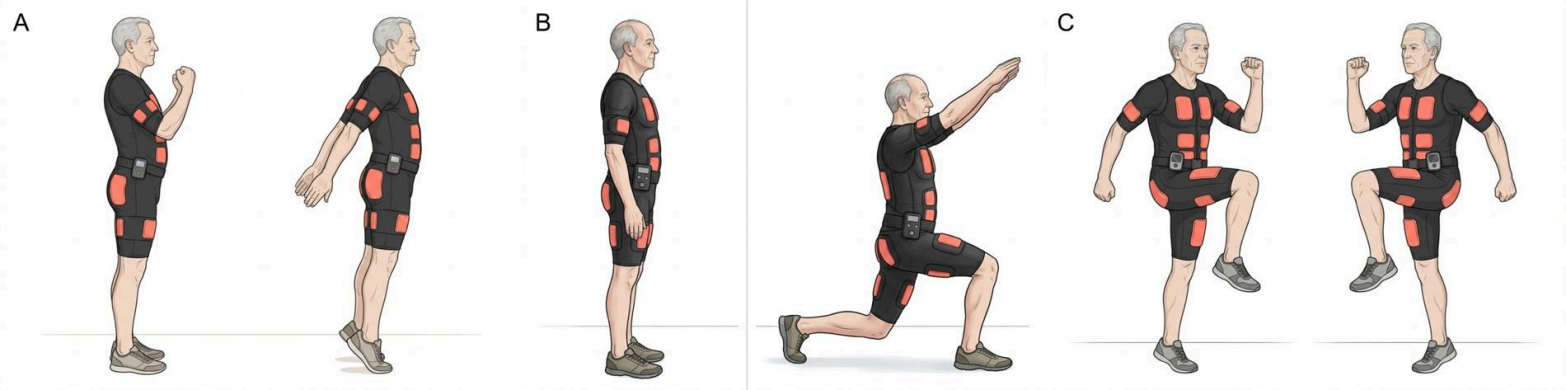
Transitional and lower-limb exercises (Exercises 4–6 of the WB-EMS protocol). **(A)** Exercise 4 — Calf raise with concurrent upper-limb extension. Standing, the patient rises onto the balls of the feet while extending the arms forward and overhead; returns to flat-foot stance with arms lowered on the “off” phase. Targets the gastrocnemius–soleus complex and shoulder girdle. **(B)** Exercise 5 — Alternating forward lunges. The patient steps forward into a controlled lunge alternately with the right and left legs. Targets the quadriceps, gluteal muscles, and hip stabilizers, recruits large lower-limb muscle mass, and adds a balance-training component. **(C)** Exercise 6 — Dynamic high-knee march. In place, the patient alternately drives each knee toward the chest at the highest tolerated height in cadence with the duty cycle. Targets hip flexors, quadriceps, and trunk stabilizers and provides a low-impact aerobic stimulus.

**Figure 4:**
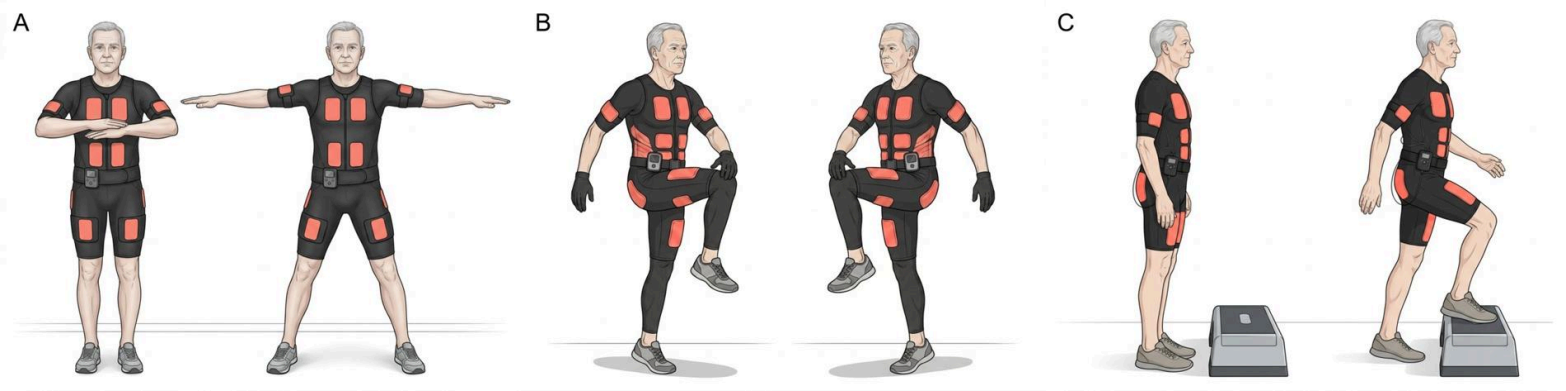
Functional multidirectional exercises (Exercises 7–9 of the WB-EMS protocol). **(A)** Exercise 7 — Lateral side-steps. From a half-squat stance the patient steps sideways with one foot then brings the other foot to meet it, returning in the opposite direction. Targets hip abductors and adductors, gluteal medius, and core stabilizers. **(B)** Exercise 8 — Cross-body knee drives. In a standing position, the patient drives one knee diagonally upward toward the opposite elbow while contracting the trunk. Targets oblique abdominal musculature, hip flexors, and adds rotational core control. **(C)** Exercise 9 — Alternating step-ups. The patient steps up onto a low platform alternately with each foot, mimicking stair climbing. Targets the quadriceps and gluteal muscles, recruits cardiovascular load, and serves as a functional close to the standing block.

WB-EMS was delivered using a Wiems Revolution Pro device (CE-marked, FDA-cleared), a wearable suit with electrodes covering the upper arms, chest, back, abdomen, gluteal region, and thighs (Figure 1A). Sessions were conducted approximately three times per week (ten sessions delivered between Session 1 and Session 10, with at least 48 h between consecutive sessions), each lasting 20 min with low-frequency bipolar impulses (85 Hz, pulse width 350 μs, duty cycle 4 s on/4 s off; i.e., a 1:1 work-to-rest ratio), with intensity titrated to the maximum tolerated level. Each session was preceded by a 5 min low-intensity warm-up on a cycle ergometer performed while wearing the suit (Figure 1B). The protocol then followed a standardized two-set structure of nine exercises separated by a two-minute rest. Each exercise lasted 45 s with a 15 s transition and was synchronized with the 4 s on/4 s off stimulation duty cycle. Exercises comprised seated movements — forward trunk inclination with upper-limb extension (Figure 2A), seated march (Figure 2B), and partial sit-to-stand (Figure 2C) — followed by transitional and lower-limb movements: calf raise with upper-limb extension (Figure 3A), alternating forward lunges (Figure 3B), dynamic high-knee march (Figure 3C); and functional multidirectional movements: lateral side-steps (Figure 4A), cross-body knee drives (Figure 4B), and alternating step-ups (Figure 4C). The full exercise protocol is provided in the Supplementary Material (Appendix S1). Protifar protein supplementation (13.2 g/day) was provided for sarcopenia prevention throughout the programme.

Because the wearable electrode suit covers the chest and back, continuous ECG monitoring during active stimulation was not feasible without risk of artefact and electrode interaction. A pre-session 12-lead ECG, continuous blood-pressure and heart-rate monitoring, and 1-, 5-, and 10 min recovery ECGs were performed for each session. A comprehensive serial safety monitoring protocol was also implemented: venous blood samples for CK, hs-TnI, NT-proBNP, CRP, and point-of-care venous blood gas with lactate were collected before and 2–3 h after each WB-EMS session.

#### 2.3.1 Rationale for the stimulation parameters

Parameter selection followed the international consensus recommendations for non-medical WB-EMS application of Kemmler et al. (7). A low-frequency setting of 85 Hz was chosen because frequencies in the 75–100 Hz range provide effective recruitment of both type I and type II fibres while remaining well tolerated and minimizing the risk of excessive muscle damage compared with the very high frequencies used in athletic high-intensity protocols. A pulse width of 350 μs was selected to ensure sufficient depolarization of deep motor units in an elderly patient with sarcopenic muscle composition, while a 4 s on/4 s off duty cycle (1:1 work-to-rest ratio) was deliberately preferred over more demanding ratios because it allows adequate intramuscular reperfusion between contractions and is the cycle most extensively validated for cardiac and frail populations. Session duration was limited to 20 min and frequency to three sessions per week with at least 48 h between sessions, in line with the consensus dose ceiling for medically supervised WB-EMS in non-athletic adults and the dose used in the only published study of supervised high-intensity WB-EMS in untrained subjects, which documented substantial muscle damage at higher Borg-rated intensities (10). Stimulation intensity was titrated session-by-session to the patient's individually tolerated submaximal level (target Borg CR-10≤ 5) rather than to maximal exertion, with the explicit aim of generating a metabolic and neuromuscular stimulus while remaining well below the rhabdomyolysis-relevant threshold.

### 2.4 Outcome measures

Primary outcomes were (a) transthoracic echocardiography with LVEF by Simpson biplane; (b) CPET on a treadmill (modified Bruce ramp protocol), reporting peak workload, VO_2_max, and arrhythmia assessment; (c) 6MWT distance; and (d) serial safety biomarkers as described above. Secondary outcomes included the EQ-5D-5L health utility questionnaire with EQ-VAS (11) and IPAQ at one-month follow-up. Functional class was assessed using the NYHA classification.

### 2.5 Safety monitoring

Cardiac monitoring during the WB-EMS sessions followed a multimodal protocol. Blood pressure (BP) and heart rate (HR) were measured before, mid-session (between sets), and immediately after every WB-EMS session, in addition to routine twice-daily ward measurements. A 12-lead electrocardiogram (ECG) was recorded immediately before and immediately after every session; continuous ECG during active stimulation was not feasible because the wet conductive textile and full-body electrode array of the WB-EMS suit mechanically interfere with surface-electrode placement and generate overwhelming stimulation artefact. A standard 12-lead ECG device was, however, kept immediately available in the exercise room throughout every session, ready to be applied without delay should any symptom, abnormal haemodynamic reading, or suspicion of arrhythmia arise. Each session was conducted with full cardiac safety equipment immediately at hand — an external automated/manual defibrillator, an emergency drug trolley, oxygen supply, suction unit, and bag-valve mask — and with at least one physician trained in advanced cardiac life support present in the unit. Outside the WB-EMS sessions, continuous in-hospital telemetric ECG monitoring was maintained 24 h/day for the full admission. Pre-defined stopping criteria (systolic BP >180 mmHg or <90 mmHg, HR > 85% of age-predicted maximum, any sustained arrhythmia, SpO_2_ <92%, Borg dyspnoea ≥7, or patient request) were never triggered during the protocol.

Serial biomarker data are summarized in the (Supplementary Table S2). CK peaked at 198 U/L after the second WB-EMS session, remaining within the normal reference range (20–200 U/L) and well below the 4× upper limit of normal safety threshold for WB-EMS-related rhabdomyolysis. CK subsequently declined to 49–69 U/L by the final monitoring timepoints, consistent with physiological adaptation. hs-TnI remained below the male upper reference limit (<17.5 ng/L) throughout the programme (range 3.2–8.7 ng/L); the two highest values (8.7 and 8.6 ng/L) were measured on the final day of the programme and were not temporally linked to a WB-EMS session, providing no evidence of WB-EMS-related myocardial injury.

Pre-exercise NT-proBNP fluctuated across the programme (range 318.3–1,019.3 pg/mL; baseline 544 pg/mL, nadir 318.3 pg/mL before Session 8, final value 1,019.3 pg/mL on the final day of the CR programme, Session 10/discharge day), rather than following a monotonic decline. The final-day rise to 1,019.3 pg/mL most likely reflects greater training effort and increased patient motivation on the last exercise day rather than clinical decompensation, an interpretation supported by the concurrently highest post-session venous lactate of the entire programme on the final day (Session 10; 4.4 mmol/L; *Δ* + 2.9 mmol/L vs. a mean *Δ* of approximately +1.5 mmol/L across the preceding nine sessions; Supplementary Table S2, Panel B). CRP declined from 5.1 mg/L at admission to values consistently <0.6 mg/L from approximately the start of CR week 2 onward, reflecting resolution of the preceding inflammatory episode, with a mild non-specific rise to 1.1–1.7 mg/L on the final day without clinical correlate. Post-exercise venous lactate ranged 2.0–4.4 mmol/L (pre-exercise 1.1–1.6 mmol/L) across the ten sessions, with venous pH stable throughout (7.37–7.47), confirming appropriate lactate kinetics without metabolic acidosis. No hypotensive episodes, significant arrhythmias attributable to WB-EMS, or musculoskeletal injuries were recorded during any session.

### 2.6 Cardiac arrhythmia management

Frequent ventricular ectopy (PVCs, bigeminy, couplets) and salvos of non-sustained ventricular tachycardia (NSVT) were identified during the index Cardiology admission and on baseline CPET (the CR admission day), where they appeared at submaximal workload, were suppressed at peak exercise, and increased during recovery. Antiarrhythmic therapy with amiodarone was therefore initiated during the index admission (intravenous 300 mg on day 9, oral loading 600 mg/day on days 7–15, then maintenance 200 mg/day from day 16) and continued throughout cardiac rehabilitation. A pre-rehabilitation Holter ECG performed in the days immediately preceding the first WB-EMS session documented marked reduction of ventricular ectopy and absence of NSVT, confirming pharmacological control before the patient entered the WB-EMS protocol. Continuous in-hospital telemetric ECG monitoring was maintained 24 h/day throughout the 24-day admission, and pre- and post-session 12-lead ECGs were obtained for every WB-EMS session. No recurrence of NSVT, sustained ventricular arrhythmia, or new clinically relevant rhythm disturbance attributable to WB-EMS was documented during any of the ten sessions or in the surrounding telemetric record. Arrhythmia burden on the discharge CPET (the day before discharge) was markedly reduced to isolated PVCs with complete suppression at peak exercise.

### 2.7 Outcomes at discharge and one-month follow-up

Discharge echocardiography (three days before discharge) demonstrated marked improvement: LVEF 54% by Simpson biplane (automated 51%), with end-systolic diameter reduced from 55 to 50 mm and end-diastolic diameter 66 mm. LV systolic function was reclassified from severely to mildly impaired. Regional wall motion abnormalities included akinesia of the basal inferior segment and hypokinesia of the apical/mid posterior, lateral, and anterior walls. Dyssynchrony persisted. Global longitudinal strain partially improved (parasternal long-axis from −5.5% to −8.6%). TAPSE improved from 23 to 26 mm; S′RV was 29 cm/s. Mitral regurgitation was qualitatively unchanged but quantitatively reduced in the setting of reverse LV remodelling and was graded as mild. New findings included mild aortic stenosis (Vmax 210 cm/s, mean gradient 10 mmHg) and a patent foramen ovale without shunt.

Discharge CPET (the day before discharge) demonstrated peak workload 97 W (67% predicted; +47% from baseline), VO_2_max 19.02 mL/min/kg (+9.2%), peak METs 5.5, and peak heart rate 130/min (88% predicted; respiratory exchange ratio 0.96). Arrhythmia burden was markedly reduced to isolated PVCs with complete suppression at peak exercise. Pressor and chronotropic responses were normal (resting blood pressure 140/80, maximum 160/90 mmHg). Resting ECG showed sinus rhythm at 83/min, diffuse intraventricular conduction delay, an old anteroseptal MI pattern, QRS 132 ms, QTc 465 ms. 6MWT distance improved from 390 to 590 m (+51%). NYHA functional class improved from III to I. The EQ-5D-5L profile normalized from 11,112 to 11,111, and EQ-VAS improved from 60 to 90/100 (+30 points).

One-month follow-up IPAQ (one month after discharge) demonstrated a substantial increase in physical activity: daily walking (seven days/week, approximately 1.5–2 h); cycling one day/week; intensive domestic activity (seven days/week, approximately 1.5 h); and moderate domestic activity (seven days/week). The patient was discharged on the final day in good general condition, without complications throughout the 24-day admission Overall, functional, echocardiographic, and quality-of-life measures improved between baseline and discharge (Table 2).

**Table 2:** Summary of clinical outcomes at baseline and discharge.

Outcome measure	Baseline	Discharge	Change
LVEF, Simpson biplane (%)	27	54	+27 pp
LV end-systolic diameter (mm)	55	50	−5
TAPSE (mm)	23	26	+3
Peak workload (W)	66 (46% pred.)	97 (67% pred.)	+47%
VO_2_max (mL/min/kg)	17.42	19.02	+9.2%
Peak METs	5.0	5.5	+0.5
6MWT distance (m)	390	590	+51%
NT-proBNP (pg/mL)	544	1,019.3 (final); 318.3 (nadir)	Fluctuated; range 318.3–1,019.3
NYHA class	III	I	−2
EQ-5D-5L profile	11,112	11,111	Normalized
EQ-VAS (/100)	60	90	+30

LVEF, left ventricular ejection fraction; LV, left ventricular; TAPSE, tricuspid annular plane systolic excursion; VO_2_max, peak oxygen consumption; METs, metabolic equivalents; 6MWT, six-minute walk test; NT-proBNP, N-terminal pro-brain natriuretic peptide; NYHA, New York Heart Association; EQ-VAS, EQ-5D-5L visual analogue scale; pp, percentage points; pred., predicted.

## 3 Discussion

This case describes the first application of WB-EMS as an adjunct to standard inpatient CR in HFrEF, demonstrating improvements across multiple assessed domains: echocardiographic function (LVEF 27% to 54%), exercise capacity (peak workload +47%, 6MWT +51%, VO_2_peak +9.2%), arrhythmia burden, and quality of life (EQ-VAS +30 points), with all safety biomarkers remaining within acceptable limits throughout the 24-day programme. Pre-exercise NT-proBNP fluctuated rather than declining monotonically; the final-day rise most likely reflected greater training effort and patient motivation on the last exercise day, supported by the concurrently highest post-session venous lactate of the entire programme.

The existing NMES literature in HF provides biological plausibility for these findings. The systematic review with meta-analysis by Gomes Neto et al. (3) demonstrated that localized NMES significantly improved peak VO_2_, 6MWT distance, quality of life, peripheral muscle strength, and endothelial function in patients with HF. Prior EMS studies in HF predominantly employed localized quadriceps or calf stimulation; the WB-EMS approach activates a larger muscle mass simultaneously across multiple groups, potentially amplifying the metabolic and haemodynamic stimuli. The companion systematic review by our group (6) confirmed that peripheral muscle electrostimulation is safe and feasible in frail elderly cardiac patients, with no cardiovascular adverse events reported across the included trials — consistent with the present findings. Kemmler et al. (7) further confirmed the efficacy and safety of WB-EMS in non-athletic adults.

The safety profile observed in this case is reassuring. CK remained below the 4× upper limit of normal safety threshold throughout, with a transient peak on day three followed by progressive decline consistent with physiological adaptation — a pattern concordant with Ono et al. (4), who reported safe improvements in physical function with home-based NMES in frail older adults with chronic HF, and with the ACTIVE-EMS trial (5). hs-TnI stability without exercise-induced rises provides no evidence of WB-EMS-related myocardial injury. Appropriate lactate kinetics (post-exercise elevation with rapid normalization and stable pH) confirm that sessions were conducted within safe metabolic limits.

The dramatic LVEF improvement from 27% to 54% warrants careful interpretation. This magnitude of recovery most likely reflects reversal of myocardial stunning following coronary revascularization combined with optimized GDMT per the 2023 ESC focused update (9) and the comprehensive CR programme (1, 2), rather than an isolated WB-EMS effect. The precise contribution of WB-EMS to the observed improvements cannot be determined from a single-arm observation and requires further investigation in a larger, controlled cohort within the ongoing randomized trial.

**Discordance between central and peripheral recovery.** A notable feature of this case is the apparent discordance between the magnitude of central cardiac recovery (LVEF 27% → 54%, a 27 percentage-point gain) and the more modest, age-appropriate improvement in peripheral exercise capacity (peak workload +47%, VO_2_max +9.2%, 6MWT +51%). According to the Fick principle, peak VO_2_ is the product of cardiac output and peripheral arterio-venous oxygen difference; in a 73-year-old patient with long-standing HFrEF, severe deconditioning, type 2 diabetes, and chronic kidney disease, peripheral oxygen extraction and skeletal-muscle oxidative capacity are major rate-limiting determinants of exercise tolerance and adapt over weeks to months rather than over a 24-day inpatient stay. In addition, Simpson-biplane LVEF is highly load-dependent: optimized GDMT (sacubitril/valsartan, eplerenone, beta-blocker, SGLT2 inhibitor, intensified diuresis) acutely reduces both pre- and afterload and unmasks contractile reserve that was previously concealed by adverse loading conditions. The observed pattern — early, large echocardiographic gains driven by revascularization, reverse remodelling and pharmacological unloading, alongside a smaller but clinically meaningful peripheral functional gain — is therefore physiologically expected. It also defines a plausible therapeutic niche for WB-EMS: an adjunctive stimulus targeting precisely the peripheral muscular component that lags behind central recovery, to be tested formally in the ongoing randomized trial.

**Why no biochemical muscle damage was observed.** The absence of clinically relevant muscle damage in this patient (CK peak 198 U/L, well within the normal reference range and far below the 4× upper-limit-of-normal threshold for WB-EMS-associated rhabdomyolysis) contrasts sharply with the existing WB-EMS muscle-damage literature in untrained subjects. Teschler and colleagues (10) reported peak CK values reaching approximately 18,358 U/L at 72 h after a single bout performed at maximal Borg ratings (≥18 on the Borg 6–20 scale). More recently, Melekoğlu and colleagues (12) demonstrated that even a single WB-EMS session performed at low perceived exertion (RPE < 13) in sedentary, EMS-naïve adults elicited statistically significant elevations in serum creatine kinase and myoglobin, peaking at 72 h at approximately 1,784 U/L and 180.6 ng/mL respectively — confirming that initial WB-EMS exposure imposes substantial physiological strain even when the session feels subjectively easy. Three features of the present protocol plausibly explain why no comparable rise was observed in our patient. First, stimulation intensity was deliberately submaximal and titrated session-by-session to a tolerated Borg CR-10 of approximately 5, rather than to maximal exertion. Second, dose was tightly capped — 20 min sessions, three times per week, with at least 48 h between sessions — in line with consensus recommendations (7), allowing full recovery of CK and inflammatory markers between exposures. Third, sessions were embedded in an active, supervised rehabilitation programme with progressive load and full cardiac monitoring, so the very first WB-EMS exposure was already preceded by several days of standard aerobic and resistance work. The combination of submaximal intensity, conservative dose, and a repeated-bout/“rest-and-recover” schedule is therefore the most plausible explanation for the absence of biochemical muscle damage, and it supports a specifically cardiac-rehabilitation–oriented WB-EMS dose that differs from the high-intensity protocols used in fitness and sports-medicine settings.

Strengths of this report include comprehensive serial biomarker monitoring before and after each session, providing granular safety data rarely available in NMES studies; use of validated patient-reported outcome measures (EQ-5D-5L (11) and IPAQ); and adherence to the CARE guidelines (8). Limitations include the single-case design, which precludes causal inference or generalizability. The short follow-up limits conclusions about durability of improvement. Major confounders include concurrent initiation of optimized GDMT, post-revascularization myocardial recovery (stunning reversal), and the multicomponent nature of the CR programme, each of which independently contributes to improvement. Results from the full randomized cohort (EMS-PL HF STUDY, planned *n* = 45) will be required to determine the independent efficacy of WB-EMS in HFrEF cardiac rehabilitation.

### 3.1 Implications for physiotherapy practice

The findings of this preliminary case report carry several implications for physiotherapy practice, pending confirmation in larger controlled studies:

- WB-EMS may represent a safe adjunctive modality in supervised inpatient cardiac rehabilitation for elderly patients with HFrEF and limited exercise tolerance, offering a complementary approach to conventional aerobic and resistance exercise training.
- A comprehensive serial biomarker monitoring protocol — including CK, hs-TnI, NT-proBNP, and point-of-care venous lactate — measured before and 2–3 h after each session provides a feasible and informative safety framework for implementing WB-EMS alongside conventional rehabilitation.
- WB-EMS simultaneously recruits multiple large muscle groups (upper limbs, trunk, lower limbs) and may offer advantages over localized stimulation in severely deconditioned patients in whom targeted exercise is insufficient to generate an adequate metabolic stimulus.
- Randomized controlled trial evidence is needed before routine clinical implementation of WB-EMS in cardiac rehabilitation for HFrEF;

### 3.2 Conclusion

In this 73-year-old patient with HFrEF undergoing inpatient cardiac rehabilitation, the addition of ten supervised WB-EMS sessions to standard CR was completed without any documented safety signal: no new or recurrent sustained arrhythmia, no significant rise in creatine kinase (peak 198 U/L) or high-sensitivity troponin I, no haemodynamic decompensation, and no skin lesions, electrode burns, or musculoskeletal injuries attributable to the stimulation. Clinical, echocardiographic, and patient-reported outcomes all improved over the 24-day programme. Because of the single-case design and the multiple concurrent therapeutic interventions (revascularization, optimized GDMT, comprehensive CR), the independent contribution of WB-EMS cannot be established here and must be confirmed in the randomized studies. The present case nonetheless supports the feasibility, tolerability, and biochemical safety of a deliberately submaximal, dose-capped WB-EMS protocol as an adjunct to conventional cardiac rehabilitation in HFrEF.

## 4 Patient perspective

The patient reported substantial subjective improvement over the course of the 24-day programme. At admission, he described limited tolerance for daily activity and rated his overall health as 60/100 on the EQ-VAS. He initially expressed mild anxiety related to the recent cardiac events but tolerated the WB-EMS sessions without discomfort, reporting that the supervised setting and incremental titration of stimulation intensity reinforced his sense of safety. By discharge, he rated his overall health at 90/100 and his EQ-5D-5L profile had normalized. At one-month follow-up, he reported walking daily for 1.5–2 h, occasional cycling, and maintaining a markedly higher level of daily physical activity than before admission, which he attributed both to recovery after revascularization and to the confidence gained during rehabilitation. The patient expressed willingness for his anonymized clinical and imaging data to be published for educational purposes.

## Data Availability

The original contributions presented in the study are included in the article/Supplementary Material, further inquiries can be directed to the corresponding author.
