# Supplementary material for: Case Report: Whole-body electrical muscle stimulation as an adjunctive tool in cardiac rehabilitation of a patient with heart failure and reduced ejection fraction
Source: Front Cardiovasc Med. 2026 Jul 8;13:1863805. doi: 10.3389/fcvm.2026.1863805 (PMC13388876; doi:10.3389/fcvm.2026.1863805)
Supplement: Supplementary file 1 [file Datasheet1.docx]

**Supplementary Material**

*Case Report: Whole-Body Electrical Muscle Stimulation as an Adjunctive Tool in Cardiac Rehabilitation of a Patient with Heart Failure and Reduced Ejection Fraction*

Sendrowski D, Polańska-Szczap A, Hus B, Vlaieva A, Markowski S, Carlé-Calo A, Kozłowski D.

This Supplementary Material accompanies the manuscript referenced above and contains: **Supplementary Table S1** (full admission-to-discharge medication table); **Supplementary Table S2** (per-session safety biomarker data); and **Appendix S1** (complete WB-EMS exercise protocol).

**Supplementary Table S1. Guideline-directed medical therapy and concomitant pharmacotherapy: status before admission, action taken during hospitalization, and dose at discharge.**

| **Drug class / Drug (INN)** | **Pre-admission** | **Action during hospitalization** | **Dose at discharge** |
| --- | --- | --- | --- |
| ARNI – sacubitril/valsartan (Entresto) | Not taken | ACEi (perindopril 10 mg q.d., reduced to 5 mg on day 3 for hypotension) switched to ARNI on day 8 | 97/103 mg b.i.d. |
| Beta-blocker – nebivolol → metoprolol succinate (Metocard ZK) | Nebivolol 5 mg q.d. | Switched to metoprolol succinate 47.5 mg b.i.d. on day 6 (HFrEF-indicated agent with mortality data) | Metoprolol succinate 47.5 mg b.i.d. (≈ 95 mg/day) |
| MRA – spironolactone → eplerenone (Espiro) | Spironolactone 25 mg q.d. | Switched to eplerenone 25 mg q.d. on day 6 (preferred MRA in post-MI HFrEF; EPHESUS evidence) | Eplerenone 25 mg q.d. |
| SGLT2 inhibitor – empagliflozin (Jardiance) | Empagliflozin 10 mg q.d. | Continued throughout hospitalization | Empagliflozin 10 mg q.d. |
| Loop diuretic – furosemide (Furosemidum) | Not taken at admission | Furosemide 40 mg i.v. on day 6 for congestion; switched to 40 mg p.o. q.d. on day 6 and maintained | Furosemide 40 mg q.d. |
| Loop diuretic – torasemide (Trifas) | Not taken | Torasemide 10 mg q.d. added on day 6 (combined loop diuretic strategy for sustained decongestion) | Torasemide 10 mg q.d. |
| Antiplatelet – ASA (Acard) | ASA 75 mg q.d. | Continued throughout hospitalization | ASA 75 mg q.d. |
| Antiplatelet – prasugrel (Efient/Bewim) | Not taken | Loading dose 60 mg p.o. on day 1 (post-PCI); maintenance 10 mg q.d. thereafter | Prasugrel 10 mg q.d. |
| Lipid-lowering – rosuvastatin + ezetimibe (Rosulip Plus) | Rosuvastatin 40 mg q.d. | Switched on day 7 to fixed-dose rosuvastatin 20 mg + ezetimibe 10 mg (ESC/EAS dyslipidaemia 2019 stepwise intensification) | Rosuvastatin 20 mg + ezetimibe 10 mg q.d. |
| Antiarrhythmic – amiodarone (Amiokordin i.v., Opacorden p.o.) | Not taken | Initiated for NSVT and frequent ventricular ectopic beats during the first days on the Cardiology Department: amiodarone 300 mg i.v. (day 9), oral loading 600 mg/day (days 7–15), then reduced to 200 mg/day (day 16); control Holter ECG without ventricular arrhythmia before initiation of the WB-EMS protocol | Amiodarone 200 mg q.d. (maintenance) |
| Calcium-channel blocker – amlodipine → lercanidipine (Lerakta) | Amlodipine 10 mg q.d. | Switched to lercanidipine 10 mg q.d. on day 9 (peripheral oedema on amlodipine); dose reduced to 5 mg q.d. on day 11 | Lercanidipine 5 mg q.d. |
| Antidiabetic – metformin (Formetic) | Not taken | Initiated 1000 mg b.i.d. on day 21 (start of Cardiac Rehabilitation Department admission) for type 2 diabetes management | Metformin 1000 mg b.i.d. |
| Proton-pump inhibitor – pantoprazole (Anesteloc) | Not taken | Pantoprazole 20 mg q.d. on day 1 (gastroprotection during DAPT) | Pantoprazole 20 mg q.d. |
| Antibiotic – ceftriaxone (Biotraxon) i.v. | Not taken | Ceftriaxone 4 g i.v. q.d. for 5 days (days 7–11) for concomitant respiratory tract infection | Discontinued before discharge |
| Antibiotic – cloxacillin (Syntarpen) i.v. | Not taken | Cloxacillin 8 g i.v. q.d. for 9 days (days 12–20) — targeted antimicrobial therapy | Discontinued before discharge |
| Anxiolytic – hydroxyzine | Not taken | Hydroxyzine 25 mg q.d. for 5 days for transient anxiety | Discontinued before discharge |
| Potassium supplementation – potassium chloride (Kaldyum) | Potassium chloride at admission | Discontinued on day 6 after K⁺ normalization following ARNI/MRA combination | Discontinued |
| Nutritional support – protein supplement (Protifar) | Not taken | Initiated on day 22 at 4.4 g three times daily (sarcopenia management during cardiac rehabilitation) | Protein supplement 4.4 g t.i.d. |

Day numbering refers to days from initial hospital admission to the Cardiology Department (day 1 = admission). Transfer to the Cardiac Rehabilitation Department occurred on day 21; discharge from the Cardiac Rehabilitation Department occurred on day 45. The WB-EMS protocol was initiated after control Holter ECG documented absence of ventricular arrhythmia. Drug names in parentheses are the commercial product names dispensed at our institution; therapeutic equivalence follows the listed INN. Source: hospital pharmacy charts of the Cardiology Department (Karta zleceń lekarskich, days 1–20) and the Cardiac Rehabilitation Department (Karta zleceń lekarskich, days 21–45). Abbreviations: ARNI, angiotensin receptor–neprilysin inhibitor; ACEi, angiotensin-converting enzyme inhibitor; ASA, acetylsalicylic acid; b.i.d., bis in die (twice daily); CR, cardiac rehabilitation; DAPT, dual antiplatelet therapy; HFrEF, heart failure with reduced ejection fraction; INN, international non-proprietary name; i.v., intravenous; MI, myocardial infarction; MRA, mineralocorticoid receptor antagonist; NSVT, non-sustained ventricular tachycardia; PCI, percutaneous coronary intervention; p.o., per os (orally); q.d., quaque die (once daily); SGLT2, sodium-glucose cotransporter 2; t.i.d., ter in die (three times daily).

**Supplementary Table S2. Per-session safety biomarker monitoring: serial CK, hs-TnI, NT-proBNP, CRP, and venous blood-gas data documented during the WB-EMS protocol.**

Venous blood samples for creatine kinase (CK), high-sensitivity troponin I (hs-TnI), N-terminal pro-brain natriuretic peptide (NT-proBNP), and C-reactive protein (CRP), together with point-of-care venous blood gas with lactate, were collected immediately before and 2–3 h after each WB-EMS session. The full WB-EMS protocol comprised ten sessions delivered from Session 1 through Session 10. Panel A summarizes the observed ranges of safety biomarkers across the admission; Panel B lists the per-session pre/post venous-lactate data documented at each WB-EMS session.

**Panel A. Observed ranges of safety biomarkers during the admission.**

| **Biomarker** | **Observed range during admission** | **Reference range** | **Safety threshold** | **Result** |
| --- | --- | --- | --- | --- |
| CK (U/L) | 49–198 (peak 198 after Session 2) | 20–200 | < 4× upper limit of normal (< 800 U/L) for WB-EMS-related rhabdomyolysis | All values within reference range; peak well below safety threshold |
| hs-TnI (ng/L) | 3.2–8.7 (final two measurements on the final day: 8.7 and 8.6 ng/L) | < 17.5 (male) | No rise > 99th percentile (URL) or > 20% change suggestive of acute myocardial injury | All values remained below the male upper reference limit (17.5 ng/L); no rise temporally linked to a WB-EMS session |
| NT-proBNP (pg/mL) | 318.3–1019.3 across the admission (baseline 544; nadir 318.3 before Session 8; final 1019.3 on the final day) | < 125 (age < 75); HF rule-in cutoff > 1800 (acute) / > 600 (chronic) for age 50–75 | Trend rather than absolute value used for interpretation | Marked early-admission decline followed by mid-protocol fluctuation; final-day rise to 1019.3 pg/mL likely reflects greater training effort and increased patient motivation on the last exercise day, supported by the concurrently highest post-session venous lactate of the entire programme (4.4 mmol/L; Δ +2.9 mmol/L on the final day — see Panel B) |
| CRP (mg/L) | < 0.6–5.1 (admission 5.1; consistently < 0.6 mid-protocol; mild final-day rise to 1.1–1.7 mg/L) | < 5.0 | No sustained acute rise | Declined from 5.1 to < 0.6 mg/L during the WB-EMS protocol; mild non-specific final-day rise without clinical correlate |
| Venous lactate (mmol/L) | Pre-session 1.1–1.6; post-session 2.0–4.4 (see Panel B for per-session values) | 0.5–1.6 | Post-exercise rise expected; absolute values < 5 indicate appropriate aerobic threshold | Appropriate post-exercise rise across all sessions; no metabolic acidosis |
| Venous pH | 7.37–7.47 | 7.32–7.42 (venous) | No acidaemia (pH < 7.30) | pH stable; no acid–base disturbance |

**Panel B. Per-session venous lactate (mmol/L) measured immediately before and 2–3 h after each WB-EMS session.**

| **Session** | **Date** | **Pre-session lactate (mmol/L)** | **Post-session lactate (mmol/L)** | **Δ Lactate** | **Adverse event** |
| --- | --- | --- | --- | --- | --- |
| 1 | Session 1 | 1.1 | 2.0 | +0.9 | None |
| 2 | Session 2 | 1.3 | 2.0 | +0.7 | None (CK peak 198 U/L within reference range) |
| 3 | Session 3 | 1.4 | 2.7 | +1.3 | None |
| 4 | Session 4 | 1.2 | 2.8 | +1.6 | None |
| 5 | Session 5 | 1.6 | 2.4 | +0.8 | None |
| 6 | Session 6 | 1.1 | 2.9 | +1.8 | None |
| 7 | Session 7 | 1.2 | 3.1 | +1.9 | None |
| 8 | Session 8 | 1.5 | 3.0 | +1.5 | None |
| 9 | Session 9 | 1.3 | 2.4 | +1.1 | None |
| 10 | Session 10 | 1.5 | 4.4 | +2.9 | None |

Stimulation parameters were identical at every session: 85 Hz bipolar, 350 μs pulse width, 4 s on / 4 s off duty cycle, 20-minute active stimulation time, intensity individually titrated to the maximum subjectively tolerated current (Borg CR-10 ≤ 6/10). All ten sessions were preceded by a 5-minute cycle-ergometer warm-up while wearing the WB-EMS suit (no active stimulation) and followed the standardized two-set structure of nine exercises described in Appendix S1. Δ Lactate is post-session minus pre-session venous lactate. Sessions were delivered approximately three times per week with at least 48 h between consecutive sessions; the inter-session interval was never < 24 h. Source: hospital laboratory information system; point-of-care venous blood gas (lactate, pH) and central laboratory assays (CK, CRP, hs-TnI, NT-proBNP) collected immediately before and 2–3 h after each session. Abbreviations: CK, creatine kinase; CRP, C-reactive protein; HF, heart failure; hs-TnI, high-sensitivity troponin I; NT-proBNP, N-terminal pro-brain natriuretic peptide; URL, upper reference limit; WB-EMS, whole-body electrical muscle stimulation.

**Appendix S1. Full WB-EMS exercise protocol.**

**Equipment**

Wiems Revolution Pro WB-EMS device (Wiemspro S.L., Málaga, Spain; CE-marked, FDA-cleared) consisting of: (i) a wearable suit with bipolar electrodes positioned over major muscle groups (upper arms, chest, back, abdomen, gluteal region, and thighs); (ii) a wireless control unit allowing independent channel adjustment for each muscle group; and (iii) a wet conductive textile lining (cotton-elastane blend) moistened with tap water prior to each session to ensure optimal electrode-skin coupling.

**Stimulation parameters**

| **Parameter** | **Value** |
| --- | --- |
| Stimulation type | Bipolar, biphasic, symmetrical, charge-balanced |
| Carrier frequency | 85 Hz |
| Pulse width | 350 μs |
| Duty cycle | 4 s on / 4 s off (1 : 1 work-to-rest ratio) |
| Ramp time | 0.5 s up / 0.5 s down |
| Intensity titration | Individually titrated at the start of each session to the maximum subjectively tolerated current (Borg CR-10 perceived exertion ≤ 6/10) for each muscle channel, with channel-by-channel adjustment |
| Total stimulation time per session | 20 min (active + rest phases combined) |
| Session frequency | Approximately three sessions per week, with ≥ 48 h between consecutive sessions |
| Programme duration | Ten sessions over approximately three weeks (Session 1 to Session 10) |

**Session structure**

Each WB-EMS session was preceded by a 5-minute low-intensity warm-up performed on a cycle ergometer while wearing the WB-EMS suit (no active stimulation during warm-up). The active stimulation phase comprised nine exercises performed in a standardized order, organized in two sets separated by a 2-minute seated rest interval. Each exercise lasted 45 s (synchronized with the 4 s on / 4 s off stimulation duty cycle) with a 15 s transition between exercises. The total session time, including warm-up and inter-set rest, was approximately 30 minutes; the active stimulation time per session was 20 minutes.

| **Phase** | **Duration** | **Activity** | **WB-EMS stimulation** |
| --- | --- | --- | --- |
| Warm-up | 5 min | Cycle ergometer at 40–50 W, cadence 50 rpm | Suit donned; stimulation OFF |
| Active set 1 | 9 min (9 × 45 s + 8 × 15 s) | Exercises 1–9 (see below) performed once each | ON during 45 s exercise; OFF during 15 s transition |
| Inter-set rest | 2 min | Seated rest; sips of water permitted | Stimulation OFF |
| Active set 2 | 9 min (9 × 45 s + 8 × 15 s) | Exercises 1–9 repeated in the same order | ON during 45 s exercise; OFF during 15 s transition |
| Cool-down | 5 min | Seated breathing/relaxation; suit doffed at end | Stimulation OFF |

**Exercises (in order)**

| **#** | **Exercise** | **Position** | **Description** | **Primary muscle groups** | **Figure** |
| --- | --- | --- | --- | --- | --- |
| 1 | Seated trunk inclination with arm extension | Seated | Forward trunk flexion to ~30° with simultaneous bilateral upper-limb extension overhead; return to upright | Erector spinae, deltoid, triceps brachii, rectus abdominis | Figure 3 |
| 2 | Seated march | Seated | Alternating hip and knee flexion (high-knee march) at a self-paced rhythm | Iliopsoas, rectus femoris, abdominal core | Figure 4 |
| 3 | Partial sit-to-stand | Sit-to-stand | Partial extension from a seated position to an upright stance and return; arms crossed over chest | Quadriceps femoris, gluteus maximus, hamstrings | Figure 5 |
| 4 | Calf raise with arm extension | Standing | Bilateral plantarflexion (heel raise) combined with simultaneous bilateral upper-limb extension overhead | Gastrocnemius, soleus, deltoid, triceps brachii | Figure 6 |
| 5 | Alternating forward lunges | Standing | Alternating forward step into a lunge position with knee flexion to ~90°; return to standing | Quadriceps femoris, gluteus maximus, hamstrings, gastrocnemius | Figure 7 |
| 6 | Dynamic high-knee march | Standing | In-place march with high-knee flexion to ~90°; opposite arm swing | Iliopsoas, rectus femoris, abdominal core, deltoid | Figure 8 |
| 7 | Lateral side-steps | Standing | Side-stepping to the right and left; arms held in athletic guard position | Gluteus medius, hip abductors, quadriceps, gastrocnemius | Figure 9 |
| 8 | Cross-body knee drives | Standing | Alternating elbow-to-opposite-knee drive (twisting); contralateral arm extension | Rectus abdominis, obliques, iliopsoas, deltoid | Figure 10 |
| 9 | Alternating step-ups | Standing | Alternating step-ups onto a low platform (~20 cm); return to floor; opposite arm swing | Quadriceps femoris, gluteus maximus, hamstrings, gastrocnemius | Figure 11 |

**Concurrent supervision and safety**

Each session was supervised by a physiotherapist trained in WB-EMS and conducted in the presence of at least one physician trained in advanced cardiac life support. The exercise room was equipped with: an external automated/manual defibrillator; a 12-lead electrocardiogram (ECG) device kept immediately available throughout every session (continuous ECG during active stimulation was not feasible due to electrode–suit interaction); an emergency drug trolley; oxygen supply; suction unit; and bag-valve mask. Blood pressure and heart rate were measured before, mid-session (between sets), and immediately after each session. A 12-lead ECG was recorded immediately before and immediately after each session. The patient was continuously monitored by telemetric ECG outside the WB-EMS sessions for the full duration of the inpatient stay. Pre-defined stopping criteria — systolic blood pressure > 180 mmHg or < 90 mmHg, heart rate > 85% of age-predicted maximum, any sustained arrhythmia, SpO_2_ < 92%, Borg dyspnoea ≥ 7, or any patient request — were never triggered during the protocol.

**Nutritional adjunct**

Protein supplementation (Protifar, 13.2 g/day) was provided throughout the cardiac rehabilitation programme for sarcopenia prevention, consistent with the European Society for Clinical Nutrition and Metabolism (ESPEN) recommendations for older adults at risk of sarcopenia.
